# Optimization of Preservation Methods Allows Deeper Insights into Changes of Raw Milk Microbiota

**DOI:** 10.3390/microorganisms8030368

**Published:** 2020-03-05

**Authors:** Alexandre J. Kennang Ouamba, Gisèle LaPointe, Simon Dufour, Denis Roy

**Affiliations:** 1 Département des Sciences des aliments, Laboratoire de génomique microbienne, Université Laval, Quebec, QC G1V 0A6, Canada; alexandre-jules.kennang-ouamba.1@ulaval.ca; 2 Regroupement FRQ-NT Op+lait, Saint-Hyacinthe, Quebec, QC J2S 2M2, Canada; glapoint@uoguelph.ca (G.L.); simon.dufour@umontreal.ca (S.D.); 3 Department of Food Science, University of Guelph, 50 Stone Road E, Guelph, ON N1G 2W1, Canada; 4 Département de Pathologie et Microbiologie, Faculté de Médecine Vétérinaire, Université de Montréal, Saint-Hyacinthe, Quebec, QC J2S 2M2, Canada

**Keywords:** raw milk microbiota, preservation method, azidiol, bronopol, dimethyl sulfoxide

## Abstract

The temporal instability of raw milk microbiota drastically affects the reliability of microbiome studies. However, little is known about the microbial integrity in preserved samples. Raw cow milk samples were preserved with azidiol or bronopol and stored at 4 °C, or with dimethyl sulfoxide (DMSO) or a mixture of azidiol and DMSO and stored at −20 °C for up to 30 days. Aliquots of 5-, 10-, and 30-day post-storage were treated with propidium monoazide (PMA), then analyzed by sequencing the 16S rRNA gene V3-V4 and V6-V8 regions. The V6-V8 gave a higher richness and lower diversity than the V3-V4 region. After 5-day storage at 4 °C, the microbiota of unpreserved samples was characterized by a drastic decrease in diversity, and a significant shift in community structure. The treatment with azidiol and DMSO conferred the best community stabilization in preserved raw milk. Interestingly, the azidiol treatment performed as well for up to 10 days, thus appearing as a suitable alternative. However, neither azidiol nor bronopol could minimize fungal proliferation as revealed by PMA-qPCR assays. This study demonstrates the preservative ability of a mixture of azidiol and DMSO and provides deeper insights into the microbial changes occurring during the cold storage of preserved raw milk.

## 1. Introduction

The interest in culture-independent approaches to study the microbiological quality of dairy milk has continued to rise, especially with the advent of high-throughput omics technologies in the field of dairy science and technology, including metatranscriptomics, metaproteomics, and metagenomics [1]. Closely related to the logistics of implementing farming practices across the dairy production chain, microorganisms found in bulk tank milk have been shown to accurately typify dairy farms and the various inherent sources of contamination [2,3]. Raw milk samples thus generally harbor diverse microbial communities of which most can easily develop due to high nutrient and water contents of the matrix. Whether they are spoilage microorganisms [4], disease causing [5], health promoting, or of technological interest [6,7], microbial dynamics is still occurring in raw milk once refrigerated immediately after milking [8,9] and during transportation to dairy processing facilities [10]. This temporal instability of milk microbiota can considerably affect sample microbial integrity, especially in prolonged sampling or when the sample delivery time to the laboratory is delayed, as it frequently happens in large-scale studies. Keeping the microbial communities in collected samples intact until processing highly contributes to guarantee the quality, validity, and reliability of the derived information. In this context, freezing biological samples at −20 or −80 °C immediately after collection has been generally accepted as the gold standard approach for preserving the microbiome profile over time [11,12,13,14]. However, the implementation and efficient management of such low temperature conditions when sampling in remote locations are hard to achieve, particularly if relatively large volumes of liquids are collected. Alternatively, several preservative chemicals have been developed to prevent milk spoilage or preserve milk constituents, among which bronopol and azidiol are commonly used for milk chemical analysis [15].

The biocidal or bacteriostatic activity of bronopol (2-bromo-2-nitropropane-1,3-diol) has been shown to depend on the initial bacterial load in raw milk, unlike azidiol (a mixture of chloramphenicol and sodium azide) which exerts bacteriostatic activity [16]. Although these preservatives have a broad spectrum of activity against Gram-positive and Gram-negative bacteria, their impact on the abundance and diversity of raw milk microbiota is not well known. Previous investigation outcomes on their ability to preserve microbial populations based on culture-independent techniques, once only limited to a few species for DNA isolation and PCR assays [17], are now expanding to high-throughput sequencing of whole communities [9,18,19,20]. However, few of these studies dealt with dairy milk, and none of them, to our knowledge, analyzed the temporal variations of viable microbial communities in refrigerated milk preserved with azidiol or bronopol, using high-throughput sequencing technologies. Furthermore, if samples were immediately frozen after collection, there would be no assurance of temperature stability over the course of sampling and sample conveyance due to prevailing environmental conditions and transportation time to the laboratory. Additionally, freezing milk samples may affect the viability of some coliforms, psychrotrophic microorganisms, or bacteria belonging to the genus *Mycoplasma* [21,22]. In a previous study on the cryopreservation of anaerobic ammonium-oxidizing bacteria, Heylen et al. [23] recommended the use of dimethyl sulfoxide (DMSO) over glycerol as cryopreservative agent to improve bacterial recovery. For viable microbiota profiling, the effectiveness of propidium monoazide (PMA) has been largely demonstrated, particularly in combination with quantitative PCR. The concept of viability-PCR was first introduced by Nogva et al. in 2003 [24] as they used ethidium monoazide in conjunction with PCR to specifically amplify DNA from viable bacterial cells (i.e., with non-compromised membrane integrity), and later by Nocker et al. in 2006 [25] for the same purpose, but with PMA instead, showing improved specificity (see reviews by Fittipaldi et al. [26] and Elizaquivel et al. [27] for more information on practical aspects and challenges associated with viability dye). If azidiol- or bronopol-treated samples are suitable for culture-independent analytical methods, no information has been reported on the suitability of PMA for use concurrently. Hence, we hypothesized that a mixture of DMSO as cryopreservative and azidiol as preservative will permit a better stability of the milk microbial composition, diversity, and viability when samples are stored at −20 °C.

**Figure 1:**
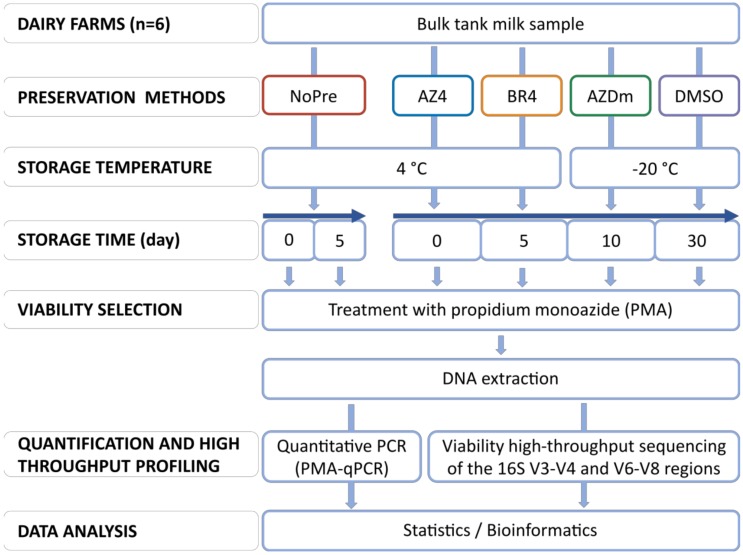
Experimental design of the study. Raw milk samples collected from six dairy farms were aliquoted and treated with four preservation methods, and then stored in a refrigerator or in a freezer according to the preservative used. Preservation included NoPre: no treatment, AZ4: Azidiol, BR4: Bronopol, AZDm: combination of azidiol and dimethyl sulfoxide, and DMSO: dimethyl sulfoxide. Preservative-free samples were stored for 5 days, and preserved samples for up to 30 days. In addition to freshly treated aliquots, sub-samples were taken from the storage after 5, 10, and 30 days, then treated with propidium monoazide and subjected to microbial analyses through quantitative PCR and 16S rRNA sequencing of the V3-V4 and V6-V8 regions. Data were analyzed to evaluate the microbiota stabilizing effects of the preservation methods as depicted by the two hypervariable regions.

In order to get more insight into the ability of preservation methods to stabilize the microbiota in raw dairy milk, we analyzed the temporal stability/instability of viable microbial communities in preserved milk samples collected from six dairy farms (Figure 1). We first compared community composition of fresh and unpreserved milk samples. We then evaluated four preservative substances including azidiol (AZ4), bronopol (BR4), DMSO, and a mixture of azidiol and dimethyl sulfoxide (AZDm). Community profiles obtained after 5, 10, and 30 days of storage at 4 or −20 °C were compared to those at day 0 and to fresh unpreserved samples. Prior to DNA extraction, samples were treated with PMA to account for cell viability. Viability-qPCR assays were performed on different bacterial groups as well as total fungi to complement community profiling derived from high-throughput sequencing of the 16S rRNA gene. Doing this, we also sought to evaluate whether 16S rRNA sequencing of the V3-V4 and V6-V8 regions provides consistent results.

## 2. Materials and Methods

### 2.1. Milk Sampling

Cow milk samples were collected from six dairy farms in Quebec, Canada. At each farm, about 1500 mL of raw milk was collected in sterilized two-liter glass bottles directly from the bulk tank, immediately after milking, and milk agitation. Prior to milk collection, the bulk tank valve was abundantly washed with tap water and approximately 100 mL of milk was discarded prior to sampling. Samples were then placed in a 4 °C electric cooler and transferred to the laboratory where they were processed the same day.

### 2.2. Preservation Treatments and Experimental Design

Each milk sample was mixed by inversion and immediately divided into 52 aliquots of 30 mL to apply the different preservative methods. Treatments included preservative-free milk (NoPre), azidiol with storage at 4 °C (AZ4), bronopol with storage at 4 °C (BR4), dimethyl sulfoxide with storage at −20 °C (DMSO), and a mixture of azidiol and DMSO with storage at −20 °C (AZDm). Azidiol was prepared as described previously [17] by dissolving 4.5 g of trisodium citrate 5,5-hydrate (VWR International, Radnor, Pennsylvanie, USA), 1.8 g of sodium azide (VWR International), 0.075 g of chloramphenicol (MilliporeSigma, Burlington, Massachusetts, USA), 0.035 g of bromophenol blue (MilliporeSigma), and 10 mL of ethanol (Greenfield Global, Toronto, Canada) in 100 mL of sterile distilled water. To obtain bronopol, a 5% (weight/vol) solution of 2-bromo-2-nitro-1,3-propanediol (VWR International) was prepared with sterile distilled water. Treatment AZ4 was achieved by adding 3.33 µL of azidiol per mL of milk aliquot (for a final concentration of 0.06 mg/100 mL). For BR4 treatment, 4 µL of bronopol solution per mL of milk aliquot was added (for a final concentration of 20 mg/100 mL). Milk treated with DMSO contained dimethyl sulfoxide (VWR International) at 5% [*v*/*v*] (22). Milk samples treated with AZDm were obtained by adding azidiol and DMSO to reach final concentrations of 0.06 mg/100 mL and 5%, respectively. Treated and preservative-free milk aliquots were vigorously shaken to homogenize the preparation. All the experiments were carried out aseptically on ice.

Treatment NoPre samples were processed at time zero and day 5 only, while all other treated aliquots were processed at time zero and kept at corresponding temperatures for 5, 10, and 30 days. All time zero treated aliquots were processed within an hour. Three replicate aliquots of each timepoint per treatment were prepared and randomly distributed in a 4 °C refrigerated incubator or a −20 °C freezer accordingly. Upon 5 days post-treatment and following timepoints, the three replicates per timepoint and per treatment were removed from the incubator or freezer, and, when available, the remaining aliquots were randomly displaced to account for temperature variations. Frozen aliquots were thawed for 2 h in a refrigerator. Aliquots of the same treatment for a given timepoint were then pooled for microbial cell recovery and genomic DNA extraction.

### 2.3. DNA Extraction and Sequencing of the 16S rRNA Gene

Treated and untreated pooled aliquots were fully homogenized, and 10 mL of each mixture were clarified with 2 mL of 500 mM ethylenediamine tetra-acetic acid [8] at pH 8.0 (Millipore Sigma) and centrifuged at 12,000× *g* at 4 °C for 15 min. Cell pellets were then washed twice with 1 mL sucrose buffer (sucrose 12% [w/v], 25 mM Tris-HCl pH 8.0). Cell pellets were treated with PMA using a lamp instrument (halogen 500 W, Ingenia Biosystems, Barcelona, Spain) as previously described [28]. PMA treated cells were kept at −80 °C until use. The protocol used for DNA extraction was adapted from that described by Quigley et al. [29] using a combination of chemical and mechanical lyses provided in the DNeasy PowerFood Microbial Kit (Qiagen, Hilden, Germany), and enzymatic lysis with lysozyme (MilliporeSigma), mutanolysin from *Streptomyces* (MilliporeSigma) and proteinase K (MilliporeSigma) with some modifications. Briefly, chemical and enzymatic lyses were achieved once by suspending cell pellets in 450 µL of warmed (heated at 37 °C instead of 55 °C as recommended for 10 min to prevent enzyme denaturation) solution MBL (provided in the extraction kit) containing 9 mg lysozyme and 50 U mutanolysin. The suspension was then incubated at 37 °C for 1 h, followed by the addition of 25 µL of proteinase K (20 mg/mL) and another 1-h incubation at 55 °C. Afterwards, mechanical lysis was performed using microbeads and a vortex adapter. This step and the remaining were all performed as described in the Qiagen instruction manual. DNA was quality checked with a NanoDrop spectrophotometer ND 1000 (Thermo Fisher Scientific, Burlington, ON, Canada) and quantified with a Qubit fluorometer 2.0 (Invitrogen., Burlington, ON, Canada), then stored at −20 °C until use.

DNA samples were submitted to the *Plateforme d’Analyses Génomiques* of University Laval (Quebec, Canada) for library preparation and 16S rRNA gene sequencing on the Illumina MiSeq platform, targeting two non-overlapping hypervariable regions namely V3-V4 and V6-V8. Primer pairs 341F (5′-CCTACGGGNGGCWGCAG-3′)/805R (5′-GACTACHVGGGTATCTAATCC-3′) and B969F (5′-ACGCGHNRAACCTTACC-3′)/BA1406R (5′-ACGGGCRGTGWGTRCAA -3′) were used to amplify the V3-V4 and V6-V8 regions, respectively.

### 2.4. Quantitative PCR

Gene copy number representing biomass variation across time of total lactic acid bacteria, total acetic acid bacteria, *Enterobacteriaceae*, *Pseudomonas* spp., total bacteria and total fungi in samples from all preservation methods were assessed by quantitative PCR (qPCR) on a ViiA7 system (Thermo Fisher Scientific). Species or group specific primer sets used for amplification are listed in Table 1. All qPCR amplifications were performed in triplicate with mixtures (10 µL) comprised of 3.6 µL UltraPure DNAse and RNAse free distilled water (Invitrogen), 5 µL PowerUp SYBR Green master mix (Thermo Fisher Scientific), 0.2 µL of each forward and reverse primer at 10 nM, and 1 µL DNA template. For each primer set, running parameters were set up as described in the corresponding reference provided in Table 1. Standard curves were constructed by using 10-fold serial dilutions of known genomic DNA concentrations extracted from single cultures of microorganisms (Table 1). For a standard curve, the gene copy number was determined with the following formula:

([DNA]° × AN)/(660 × NT)(1)

where [DNA]° is the DNA concentration (g/L), “AN” the Avogadro number (6.02 × 10^23^ mol^−1^), “660” the average molar weight of a base pair (g/mol/base pair), and “NT” the amplicon length (base pair). Sample microbial biomass expressed in gene copy number per milliliter of milk was calculated based on each standard curve’s equation.

### 2.5. 16S rRNA Gene Bioinformatics and Data Analysis

Adapter and primer removal from demultiplexed sequences were performed using Cutadapt (version 2.3) tool [37]. Reads corresponding to the V3-V4 and V6-V8 hypervariable regions were separately analyzed following the DADA2 (version 1.12) pipeline [38] in R environment. For both datasets, taxonomy was assigned to the resulting amplicon sequence variants (ASVs) using the Silva version 132 DADA2-formatted reference databases. Sequence alignment was performed using the DECIPHER (version 2.12.0) package [39] and the phangorn (version 2.5.3) package [40] was used to construct the phylogenetic tree as previously described [41]. Processed sequences were then imported into the phyloseq (version 1.28.0) package [42] for downstream analyses of bacterial communities for which ASVs were identified at least at the phylum level and occurred in at least two of the six fresh milk samples (i.e., NoPre treatment at day 0) with greater than 0.001% relative abundance.

We compared the V3-V4 and V6-V8 bacterial profiles by calculating corresponding Chao1, Shannon and Inverse Simpson indices on fresh milk samples using the phyloseq package. To visualize community composition and diversity variation between both hypervariable regions, heat trees (i.e., cladogram allowing the visualization of differential abundance and diversity of taxonomic data) were constructed using the Metacoder (version 0.3.2) R package [43]. A custom function was implemented in R in order to combine the two datasets. Significant differences between taxa median proportions were calculated using a Wilcoxon rank sum test on a sub-dataset composed of the core microbiota identified for both regions. Only significant features obtained after FDR (false discovery rate) correction of *p*-values were considered. Heat trees were also constructed for taxa specific to each region. A Venn diagram comparing the number of taxa identified by both regions at the genus level was constructed using an online tool (http://bioinformatics.psb.ugent.be/webtools/Venn/).

To characterize microbial alteration over time in milk without preservatives, we compared community composition and structure of 5-day stored milk with fresh samples. Besides the above-mentioned alpha-diversity measures, beta-diversity was assessed by plotting principal coordinate analysis (PCoA) with weighted and unweighted UniFrac distance metrics [44]. For alpha- and beta-diversities, a Wilcoxon rank sum test with FDR correction was performed using the ggpubr (version 0.2) R package [45]. Biomarkers distinguishing fresh from 5-day stored raw milk samples were identified by applying the linear discriminant analysis effect size (LEfSe) algorithm [46] available in the Galaxy platform [47] with default parameters. Differential abundance of taxa between both conditions and data visualization were performed using a combination of R packages phyloseq, ggplot2 version 3.1.1 [48] and DESeq2 version 1.24.0 [49].

The effect of preservation chemicals on the microbiota upon contact with milk samples was evaluated by analyzing variation in alpha- and beta-diversities before and immediately after preservatives were added to milk aliquots. Alpha-diversity measures including Chao1 and Shannon indices were computed. Differential abundance of taxa between fresh and preserved samples was calculated, and community composition, taxa prevalence, and distribution among treatments were visualized using a heat map constructed with the R package ComplexHeatmap 2.1.0 [50].

The ability of preservation methods to maintain stable milk microbiota over time was assessed by linear mixed-effects (LME) modelling using the R package nlme 3.1-140 [51]. Computing variation of diversity metrics across time was adapted from a previously described approach [52]. Briefly, models were fitted based on log- or arcsine-transformed data for variance stabilization or normality prerequisite. The random effect for the intercept and the slope was defined by dairy farm. Computed alpha-diversity measures included Chao1, Shannon, and Inverse Simpson indices. Beta-diversity trends and instability (i.e., distance variation within communities for consecutive timepoints) were evaluated using Jensen–Shannon divergence as well as weighted and unweighted UniFrac distances. We used the function emmeans() with FDR correction of *p*-values implemented in the R package emmeans 1.3.5 [53] for multiple comparison tests among storage timepoints. For beta-diversity trends, significance of the linear model was evaluated with a permutation test. PCoA was also performed on weighted UniFrac distance to estimate and visualize differences in microbial community structure over time for each preservation method. Significance of differences between communities at the four timepoints was evaluated using a Kruskal–Wallis test implemented in the R package ggpubr.

Intraclass correlation coefficients (ICC) with a one-way model was also used to assess microbial temporal stability at different taxonomic levels in preserved and non-preserved milk aliquots. We used the function icc() of the R package irr 0.84.1 [54] as previously described [55] to compute single score ICCs as an index of interrater reliability of preserved and non-preserved milk microbial community abundances over time. For each timepoint and preservation method, the calculated index quantifies abundance variability for a given taxonomic level among samples from different farms, taken as biological replicates. Intraclass correlation calculations were performed using default parameters. Their values range between 0 and 1. For ICC values less than 0.5, or between 0.5 and 0.75, or between 0.75 and 0.9, or above 0.9, they describe poor, moderate, good, or excellent reliability, respectively [56].

Taxa differential abundances between consecutive and non-consecutive timepoints were computed using the DESeq2 algorithm and feature dynamics (i.e., for each preservation method, variations in abundance and prevalence across time of taxa that underwent, at least once, a log2-fold change) were visualized with a heatmap.

## 3. Results

### 3.1. High-Throughput Sequencing of the 16S rRNA Hypervariable Regions V3-V4 and V6-V8 Reveals Different Taxonomic Profiles

Sequence processing with the DADA2 pipeline resulted in 1,882,615 reads with an average of 17,432 per sample for the V3-V4 region, whereas for the V6-V8 region, 3,555,435 reads with an average of 32,921 per sample were obtained. From these reads, 712 and 977 ASVs were inferred for V3-V4 and V6-V8 regions, respectively.

**Figure 2:**
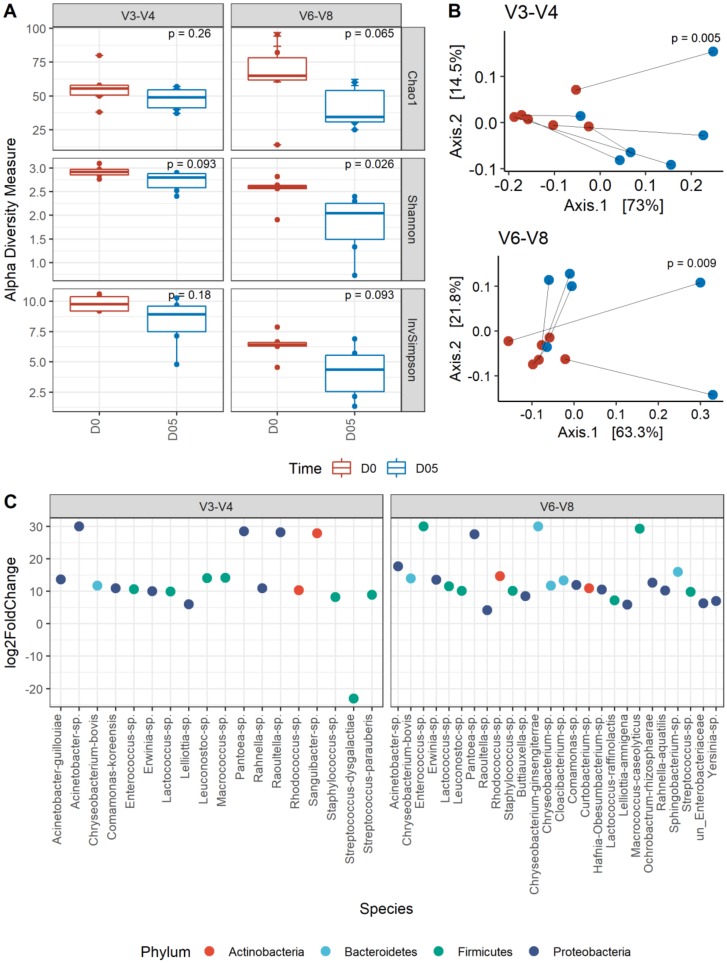
Changes in the microbial diversity of unpreserved raw milk samples. (**A**) Alpha-diversity measures of fresh (D0) and 5-day stored (D05) raw milk based on V3-V4 (left) or V6-V8 (right) datasets. *p*-values indicate the significance of differences between timepoints obtained from the Wilcoxon rank sum test. (**B**) Principal coordinate analysis on weighted UniFrac distances showing shifts in each unpreserved raw milk sample between fresh (red) and 5-day stored (blue) based on V3-V4 (top) and V6-V8 (bottom) datasets. *p*-values indicate the significance of differences between timepoints obtained from the Kruskal–Wallis test. (**C**) Visualization of species level log2-fold changes in relative taxa abundance between day 0 and day 5 based on V3-V4 (left) and V6-V8 (right) datasets. Species are colored by corresponding Phylum.

We compared the taxonomic profiles resulting from 16S rRNA sequencing of V3-V4 and V6-V8 regions in unpreserved milk samples at day 0. Consistently with the number of ASVs, the estimated richness (Chao1 index) was lower for the data from the V3-V4 region compared to the profiles obtained from the V6-V8 region (Figure 2A). However, microbial communities appeared more diverse and evenly distributed (higher Shannon and Inverse Simpson indices) with the V3-V4 than the V6-V8 region.

**Figure 3:**
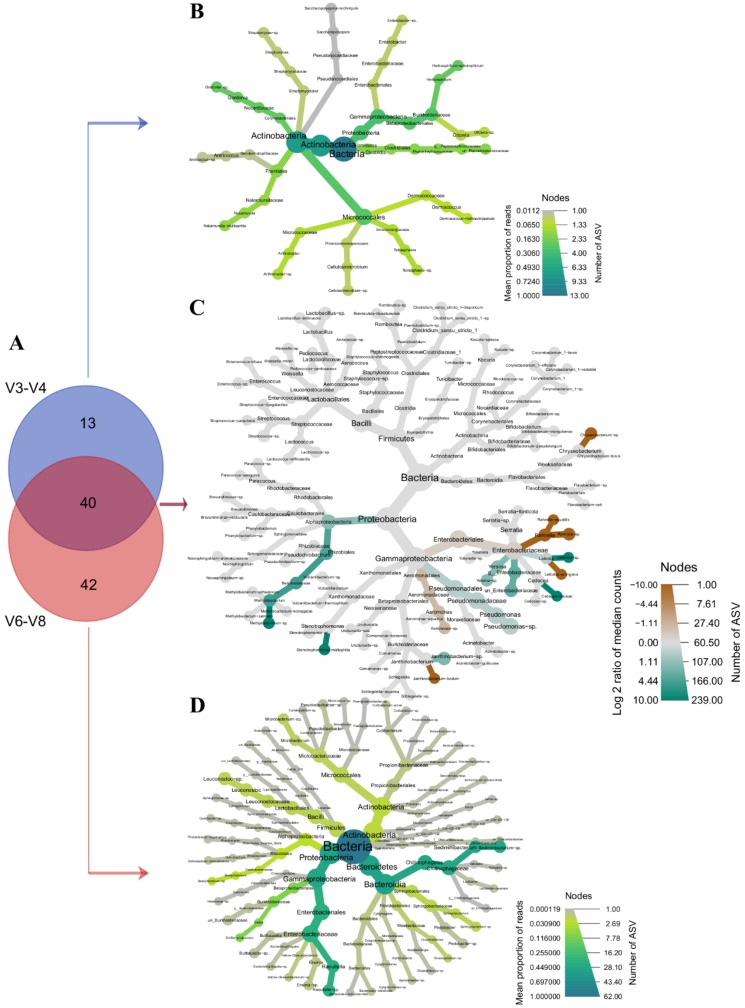
Core and differential microbial profiles obtained by partial sequencing of the 16S rRNA gene. (**A**) Venn diagram of taxa detected by sequencing the hypervariable regions V3-V4 and V6-V8. Values indicate the number of shared (purple) and uniquely identified (blue or salmon) taxa at the genus level. (**B**) Heat tree illustrating the microbial profile from taxa uniquely detected by V3-V4 sequencing. The color indicates the mean proportion of reads and the node size the number of ASV detected. (**C**) Differential abundance in the core microbiota of the V3-V4 and V6-V8 datasets. Taxa colored green are significantly abundant (*p* < 0.05) in the V3-V4 dataset, while those colored brown are significantly abundant (*p* < 0.05) in the V6-V8 dataset. The node size indicates the number of ASV. (**D**) Taxonomic distribution of taxa detected only in the V6-V8 dataset. The color and node size have, respectively, the same meaning as for (**B**).

Overall, 53 genera were identified for the V3-V4 region, compared to 82 for V6-V8, both regions sharing 40 genera (Figure 3A). Among the 13 taxa identified only in the V3-V4 region, *Actinobacteria* (*Micrococcales*, *Corynebacteriales*, *Frankiales*) and *Proteobacteria* (*Betaproteobacteriales*) were the most abundant, most of ASVs being distributed among *Actinobacteria* and *Proteobacteria* (Figure 3B). As shown in Figure 3C, the core microbiota down to the species level (i.e., species identified in both regions) was dominated by the phyla *Proteobacteria* and *Firmicutes*, while *Actinobacteria* and *Bacteroidetes* contained fewer ASVs. At the genus level, the 10 most abundant taxa for the V3-V4 region included *Serratia*, *Pseudomonas*, *Lelliottia*, *Lactococcus*, an unidentified *Enterobacteriaceae*, *Cedecea*, *Aeromonas*, *Yersinia*, *Janthinobacterium*, and *Kocuria*. Except for the genus *Lactococcus* that was not identified in one sample, these genera together with *Methylobacterium* were also the most prevalent. However, the list of the top 10 genera for the V6-V8 region was not the same, as it consisted of *Serratia*, *Pseudomonas*, *Rahnella*, *Sediminibacterium*, *Lelliottia*, *Cedecea*, *Aeromonas*, *Lactococcus*, *Raoultella*, and *Janthinobacterium*. These genera together with *Yersinia* and *Delftia* were among the most prevalent. Within the core microbiota, differentially abundant taxa were identified. They include *Methylobacterium* sp., *Stenotrophomonas maltophilia*, *Janthinobacterium* sp., *Pseudomonas* sp., *Cedecea davisae*, *Lelliottia* sp., *Yersinia* sp., and an unidentified *Enterobacteriaceae* that were significantly more abundant (*p* < 0.05) in V3-V4 sequence profiles, and *Janthinobacterium lividum*, *Aeromonas* sp., *Lelliottia amnigena*, *Rhanella* sp., *Rhanella aquatilis*, and *Chryseobacterium* sp. that were significantly more abundant (*p* < 0.05) in the V6-V8 dataset. Of the 42 uniquely identified taxa from the V6-V8 profiles, *Proteobacteria* (*Enterobacteriales*, *Betaproteobacteriales*) and *Bacteroidetes* (*Chitinophagales*) were the most abundant (Figure 3D). Most of ASVs were assigned to *Proteobacteria*, *Actinobacteria*, and *Bacteroidetes* phyla.

### 3.2. Short-Term Storage of Unpreserved Milk Resulted in Significant Alteration of the Microbial Community Structure

Regardless of the sequenced hypervariable region, the estimated richness (Chao1) and diversity metrics (Shannon and InvSimpson) significantly decreased (*p* < 0.05) after 5 days of storage at 4 °C when no preservatives were used (Figure 2A). This indicates a drastic loss of diversity and thus the emergence of a few dominant species. Consequently, a significant shift (*p* ≤ 0.008 for V3-V4, *p* ≤ 0.02 for V6-V8) in community structure occurred during storage (Figure 2B). In order to identify taxa that underwent significant increase or decrease in abundance and thus could explain the observed changes in community structure, we performed differential abundance analyses between fresh and stored milk samples without preservatives. As shown in Figure 2C which illustrates fold changes for the V3-V4 region, *Streptococcus dysgalactiae* was reduced more than 20-fold in abundance at 5 days post-storage, while the 17 other bacterial groups changed by more than five-log2. *Chryseobacterium bovis*, *Pantoea* sp., *Raoultella* sp., and *Sanguibacter* sp. had the highest (>25) log2-fold change. Most of these bacteria, particularly species of *Lactococcus*, *Acinetobacter*, *Rahnella*, *Pantoea*, *Enterococcus,* and *Lelliottia* became predominant after 5 days of storage (Figure S1). For the V6-V8 region on the other hand (Figure 2C), *Raoultella* sp. had a less than 5-fold increase in abundance, whereas the majority of the 25 other taxa exhibited between 5- and 20-fold increases in abundance. *Chryseobacterium ginsengiterrae*, *Enterococcus* sp., *Macrococcus caseolyticus,* and *Pantoea* sp. exhibited the highest (>25) fold changes. As for V3-V4 sequencing, most of these taxa, specifically species of *Lactococcus*, *Sphingobacterium*, *Acinetobacter*, *Yersinia*, *Erwinia*, and *Rahnella aquatilis* were predominant in stored milk (Figure S2). These observations are consistent with LEfSe on V3-V4 sequences that reveals *Lactococcus*, *Yersinia*, *Enterococcus*, *Macrococcus,* and *Leuconostoc* as biomarkers of 5-day stored milk without preservative at 4 °C (Figure S3A). For the V6-V8 region, only *Acinetobacter*, *Macrococcus,* and *Rhodococcus* were identified as discriminatory (Figure S3B).

### 3.3. Impact of Preservative Chemicals on the Microbiota Once Mixed with Milk

**Figure 4:**
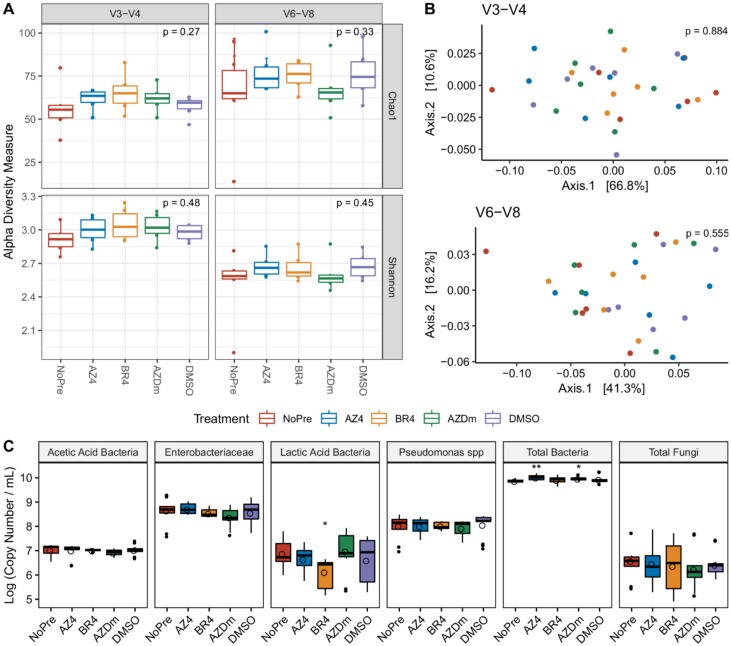
Changes occurring in the microbial composition and structure of raw milk once subjected to preservatives. Treatments include NoPre (untreated raw milk), AZ4 (raw milk treated with azidiol), BR4 (Bronopol-treated raw milk), AZDm (raw milk treated with a mixture of azidiol and dimethyl sulfoxide), and DMSO (raw milk treated with dimethyl sulfoxide). (**A**) Visualization of alpha-diversity measures following treatments as described in the V3-V4 (left) and V6-V8 (right) datasets. (**B**) Principal coordinate analysis on weighted UniFrac distances based on the V3-V4 (top) and the V6-V8 (bottom) datasets. (**C)** Boxplot showing the bacterial and fungal quantification by PMA-qPCR. Asterisks above boxes indicate a significant difference compared to NoPre, obtained by performing a Wilcoxon rank test. *p*-values are flagged with asterisks as follows: *, *p* < 0.05; **, *p* < 0.01.

In order to assess the stabilizing properties of preservation methods, we first analyzed their effect on microbial communities once mixed with fresh milk. Regardless of the sequenced region, neither the alpha-diversity metrics, including Chao1 estimates and Shannon index, nor the beta-diversity measures, including unweighted- (Figure S4A) and weighted-UniFrac distances, were significantly different before and immediately after addition of preservatives (Figure 4A,B). However, log2-fold increases in taxa abundance were observed in the V3-V4 dataset for treatments AZ4 (*Rothia*, *Leuconostoc* and *Clostridium_sensu_stricto_1*), BR4 (*Clostridium_sensu_stricto_1*), and AZDm (*Clostridium_sensu_stricto_1*), while for the V6-V8 sequenced region, only the genus *Cloacibacterium* exhibited log2-fold reduction in abundance for treatment AZ4 (Figure S4B,C). To further investigate the extent to which microbial communities were sensitive to preservative chemicals, we also performed qPCR assays to quantify and compare community gene copy numbers between treated and untreated samples immediately after treatment application. Preservative chemicals tended to increase viable bacterial load (total bacteria group), with significant differences found for treatments AZ4 (*p* < 0.01) and AZDm (*p* < 0.05) compared to NoPre (Figure 4C). No significant differences were found for any treatment for acetic acid bacteria (AAB), *Enterobacteriaceae*, and *Pseudomonas* sub-groups. Unlike other bacterial sub-groups, lactic acid bacteria (LABs) appeared more sensitive to the chemicals, with a significant decrease (*p* < 0.05), close to a log of their viable load under the effect of treatment BR4. Fungal sensitivity to preservative chemicals was also tested, for which biomass quantities in treated samples were comparable to those without treatment (NoPre), although within-sample variability was higher for BR4 and AZ4 treatments (Figure 4C).

### 3.4. Impact of Preservation Methods on Microbial Community Stability over Time

**Figure 5:**
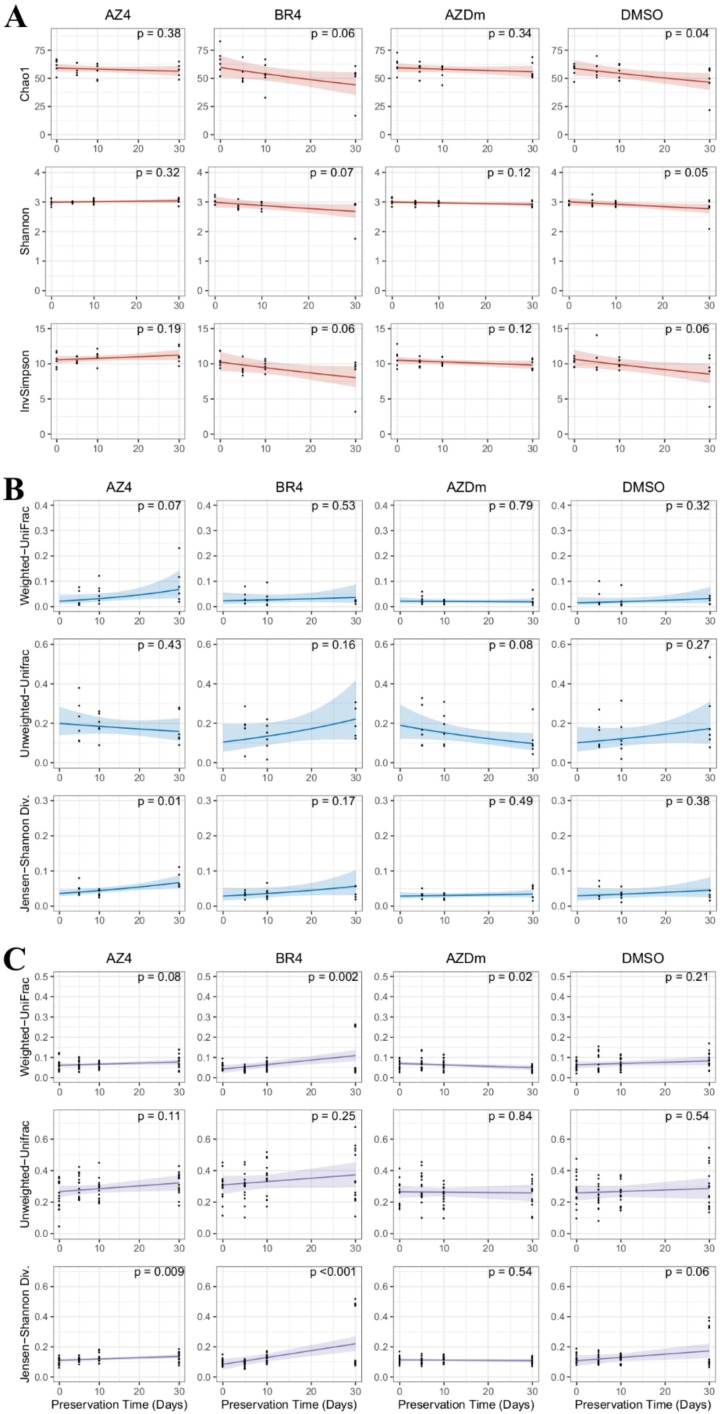
Temporal stability of microbial communities in preserved raw milk across 30 days of storage. Treatments include NoPre (untreated raw milk), AZ4 (raw milk treated with azidiol), BR4 (Bronopol-treated raw milk), AZDm (raw milk treated with a mixture of azidiol and dimethyl sulfoxide), and DMSO (raw milk treated with dimethyl sulfoxide). (**A**) Variations in alpha-diversity measures. For Chao1 estimates, Shannon and InvSimpson indices, salmon lines represent linear mixed-effects fit against the storage time and the 95% confidence interval is shaded. (**B**) Diversity trends between samples of the same farm at consecutive timepoints. Blue lines represent linear mixed-effects fit against the storage time and the 95% confidence interval is shaded. (**C**) Diversity trends between sample aliquots from different farms at consecutive timepoints. Purple lines represent linear mixed-effects fit against the storage time and the 95% confidence interval is shaded.

Following the assessment of the preservatives’ effects on the microbiota upon addition to milk, microbial community stability was evaluated at four timepoints over 30 days by comparing community diversity and structure between fresh and stored samples. As higher discriminative power was obtained with the V3-V4 based dataset, only V3-V4 figures are presented in this section; all V6-V8 homologues are available as supplemental materials. As shown in Figure 5A, no significant change in alpha-diversity measure was observed during the storage of treated samples, except the Chao1 estimate (*p* = 0.04) for DMSO treatment. However, we found no significant difference after performing a post-hoc test accounting for the multiple comparisons made (Figure S5A). Consistent results were also obtained with the V6-V8 based dataset, with no significant difference found for any of the alpha-diversity metrics, nor preservation methods (Figure S6A).

The ability of preservation methods to stabilize milk microbial communities was also evaluated by measuring Jensen–Shannon divergence, weighted- and unweighted-UniFrac distance discrepancies between serial timepoints for each single sample. Accordingly, data were recorded at three timepoints (Figure 5B). Only Jensen–Shannon divergence in treatment AZ4 showed a significant difference in distance measure between consecutive timepoints (*p* = 0.01). Most aliquots in other conditions remained relatively stable with little changes over time, although regression across timepoints may show more or less pronounced trends. Concerning Jensen–Shannon divergence measures on treatment AZ4, a post-hoc test revealed that overall milk community structure at 5 days post-storage was comparable to those at 10 and 30 days, while the 10- and 30-day samples were significantly different from one another (Figure S5B). Similar results were obtained with the V6-V8 sequence dataset regarding Jensen–Shannon divergence (Figure S6B), except that post-hoc analysis following significance found in treatment AZ4 did not reveal significantly different community structures across timepoints (Figure S7A).

Using the same distance measures as for evaluating single sample microbial stability over time, we investigated the stabilizing effects of preservation methods on community structures by comparing between-sample diversity across timepoints. Significant shifts in community structure were observed in treatments BR4 (*p* = 0.002) and AZDm (*p* = 0.02) when Weighted-UniFrac distance was used (Figure 5C). For treatment BR4, pairwise phylogenetic distances tended to increase over storage time, while for treatment AZDm, the opposite was observed, indicating for the latter a better control on microbial dynamics. This is consistent with the post-hoc test that showed no significant difference across timepoints for AZDm treated samples, while for BR4 treated samples, community structure at day 30 was significantly different from all preceding timepoints (Figure S5C). Considering unweighted-UniFrac measure, there was no significant difference in any of the four treatments, implying relatively stable phylogenetic diversity over time. Jensen–Shannon divergence showed that significant differences in community structure arose during storage in AZ4 (*p* = 0.009) and BR4 (*p* < 0.001) treated milk (Figure 5C). As illustrated in Figure S5C, these results were mostly explained by a significant difference between community structure at day 30 compared to day 0 for AZ4, and between days 0, 5, and 10 for BR4. Unlike alpha-diversity and within sample stability over time, beta-diversity analyses on the V6-V8 related dataset showed different results (Figure S6C). Using weighted-UniFrac distance, significant shifts in microbial communities were found for AZDm (*p* ≤ 0.004) and DMSO (*p* = 0.02) treated samples over time, while with unweighted-UniFrac, significant changes (*p* = 0.03) in community composition were found in AZDm treated samples only. For Jensen–Shannon divergence, significant changes (*p* = 0.01) were found for AZDm treated samples. Despite these results, based on regression lines across timepoints, there was no obvious trend in community dynamics for any treatment and distance method. However, post-hoc analyses performed on weighted-UniFrac distance distinguished community structures at different timepoints in AZDm and DMSO treatments, but not in AZDm considering unweighted-UniFrac and Jensen–Shannon measures (Figure S7B).

**Figure 6:**
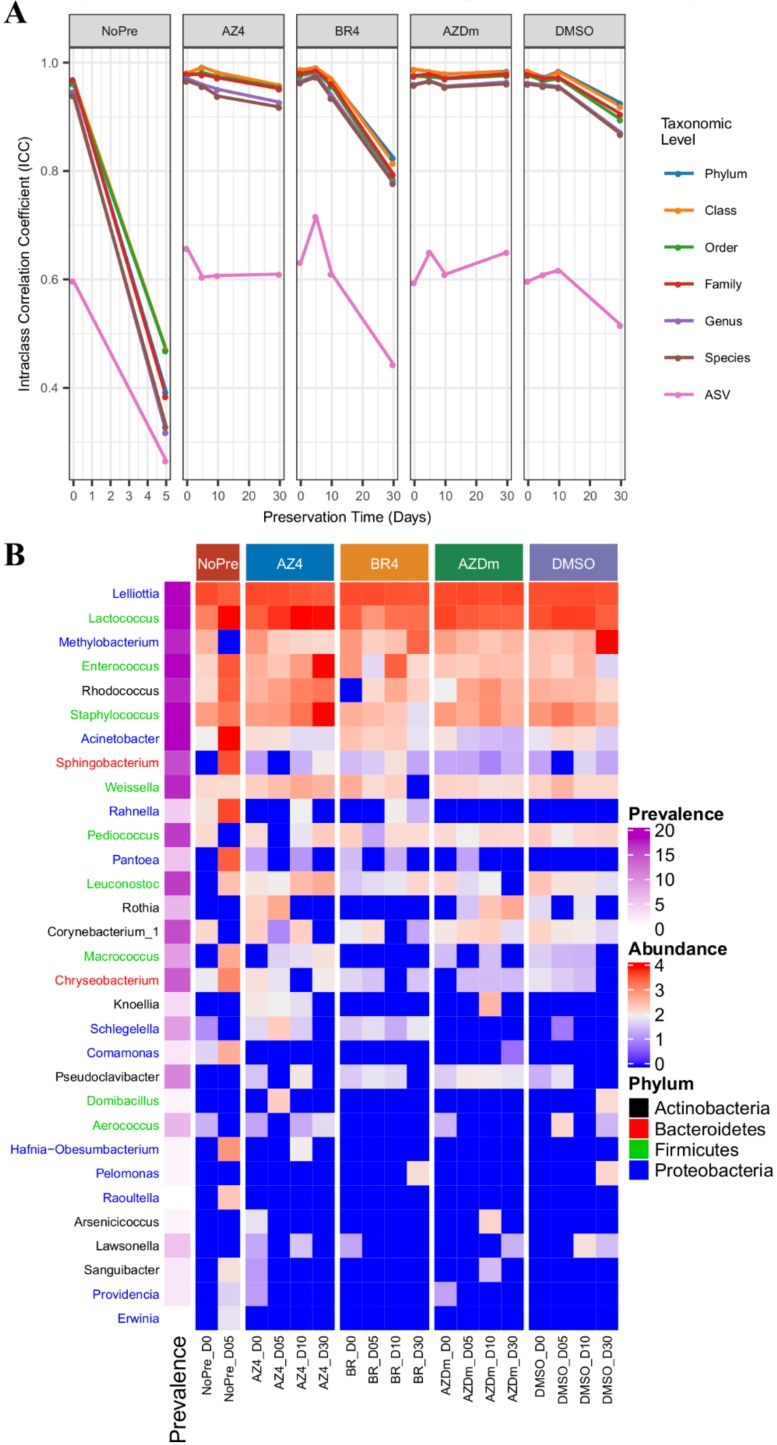
Community instability and taxa dynamics in unpreserved and preserved milk samples over storage time. Treatments include NoPre (untreated raw milk), AZ4 (raw milk treated with azidiol), BR4 (Bronopol-treated raw milk), AZDm (raw milk treated with a mixture of azidiol and dimethyl sulfoxide), and DMSO (raw milk treated with dimethyl sulfoxide). (**A**) Intraclass correlation coefficients computed for all the taxonomic levels between samples of different farms and plotted against the storage time. (**B**) Prevalence and abundance of taxa that underwent ≥ log2-fold changes. Each taxon at the genus level is colored by its corresponding phylum.

Temporal changes in microbial composition at selected taxonomic levels were evaluated by quantifying consistency in taxa relative abundance through the calculation of ICC values at each timepoint for preserved and unpreserved samples. The rationale for treatment NoPre was to demonstrate variation in bacterial abundance that occurs in the absence of any preservation measure other than storage at 4 °C. Intra class correlation results showed that for treatment NoPre, abundance consistencies at phylum down to genus levels varied from near excellent (≈0.9) to poor (<0.5) after only 5 days of storage, while at the ASV level, consistency decreased from moderate (≈0.6) to poor (<0.3), indicating drastic alteration of milk microbiota integrity during storage without preservatives (Figure 6A). No such extent of microbiome community denaturation was noticed in treated samples, except for treatment BR4 where consistency at the ASV level dropped from good (≈0.71) at day 5 to poor (≈0.42) at day 30, while remaining good despite temporal instability (ICC values ranging from ≈0.9 at day 5 to ≈0.8 at day 30) at higher taxonomic levels. Treatment AZDm exhibited the most stable consistency in taxa abundance overall, followed by treatments AZ4 and DMSO. Contrasting results were obtained for the V6-V8 sequences dataset, where ICC values across time for all treated samples remained above 0.8 (good consistency) from phylum to species level, and above 0.6 (moderate consistency) at the ASV level, with no ICC drop of more than 0.2 (Figure S8A).

To identify taxa specifically at the genus level whose changes in abundance across all consecutive timepoints may explain observed community shifts, we performed DESeq2-based differential abundance analysis. Among the 15 most abundant taxa, *Proteobacteria* including *Lelliottia*, *Methylobacterium*, *Acinetobacter*, *Rahnella*, and *Pantoea*, *Firmicutes* including *Lactococcus*, *Enterococcus*, *Staphylococcus*, *Weissella*, *Leuconostoc*, and *Macrococcus*, and to a lesser extent *Actinobacteria* including *Rhodococcus*, *Rothia,* and *Corynebacterium*_1 were the main drivers of community structure instability in preserved and unpreserved milk samples (Figure 6B). Genera exhibiting the highest log2-fold changes include *Lactococcus*, *Enterococcus,* and *Staphylococcus* for treatment AZ4, and *Methylobacterium* for treatment DMSO. Considering the V6-V8 based dataset on the other hand, the top 15 most abundant taxa explaining structural changes in microbial community over time were largely dominated by *Proteobacteria* (Figure S8B). However, genera showing the highest log2-fold change include *Lactococcus*, *Staphylococcus,* and *Enterococcus* for treatment AZ4.

**Figure 7:**
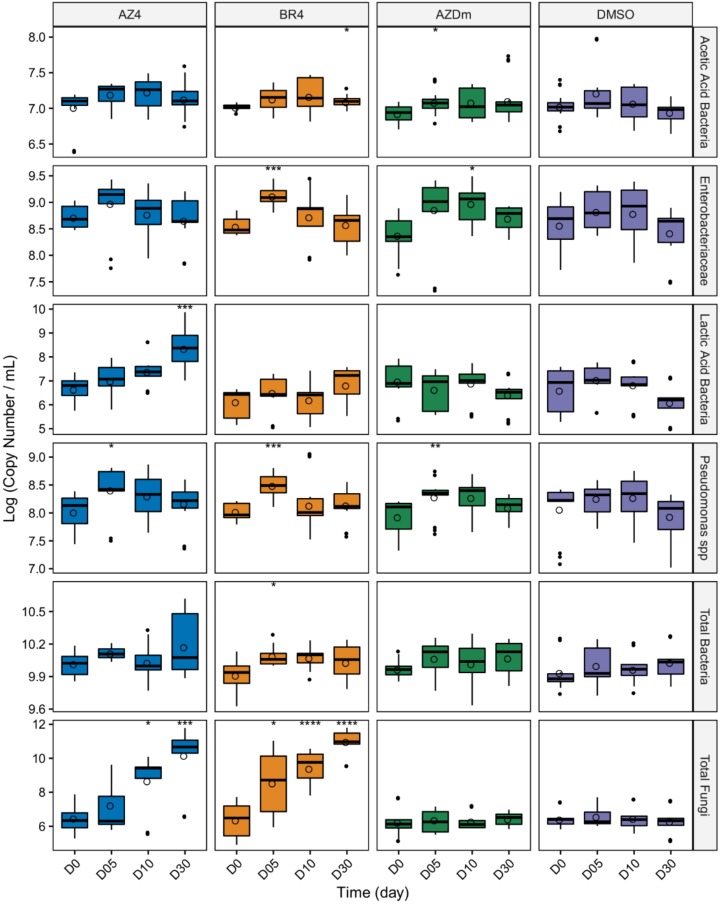
Quantification of viable microbial groups/sub-groups over time. Log copy numbers/mL are plotted against the preservation time. Copy numbers calculated at timepoints D05, D10, and D30 are compared to that at D0 using a Wilcoxon rank test. Asterisks above boxes indicate significant differences compared to D0, and flag *p*-values as follows: *, *p* < 0.05; **, *p* < 0.01; ***, *p* < 0.001; ****, *p* < 0.0001.

As a complementary approach to high throughput sequencing based analysis of microbial temporal stability, we performed PMA-qPCR to quantify variation in abundance of selected taxonomic groups over time. Viable microbial loads for taxonomic groups including AAB, *Enterobacteriaceae*, LABs, *Pseudomonas*, total bacteria, and total fungi, were expressed in gene copy number per mL of milk sample. AAB average loads were relatively stable over time for all treatments, although significant differences (*p* < 0.05) were found at days 30 for treatment BR4 and 5 for treatment AZDm compared to day 0, respectively (Figure 7). Unlike AAB communities, *Enterobacteriaceae* were slightly less sensitive to preservatives, particularly at days 5 for treatment BR4 and 10 for treatment AZDm where significant increases in bacterial loads (*p* < 0.001, *p* < 0.05, respectively) were observed. LAB were less sensitive to treatment AZ4, for which significant increases in community loads were observed at day 30 compared to day 0. For the *Pseudomonas* group, significant increases in community loads were noted at day 5 for treatments AZ4, BR4, and AZDm (*p* < 0.05, *p* < 0.001, *p* < 0.01, respectively). Overall, viability-PCR for total bacteria showed that communities were relatively stable for all treatments, except treatment BR4 for which a significant increase in bacterial load was observed at day 5, even though variation amplitudes were only a few tenths of a log. On the other hand, fungal communities were stable under preservation methods involving freezing (AZDm and DMSO) but grew dynamically under treatments AZ4 and BR4, which both involved refrigeration at 4 °C. Compared to day 0, fungal loads significantly increased at days 10 and 30 (*p* < 0.05, *p* < 0.001, respectively) for treatment AZ4, and days 5, 10, and 30 (*p* < 0.05, *p* < 0.0001, *p* < 0.0001, respectively) for treatment BR4.

## 4. Discussion

In the current study, the “viability high-throughput sequencing” approach was used to assess the efficiency of raw milk preservation methods. By analogy to viability-PCR, “viability high-throughput sequencing” refers to high-throughput sequencing of DNA from live microbial cells as depicted by PMA. Regarding technical limitations associated with the use of PMA [26], a previous study conducted in our laboratory [28] has optimized the application of PMA-qPCR on milk and cheese samples, and since then it has become routine practice on various matrices [57,58,59,60]. We showed that V3-V4 and V6-V8 hypervariable regions of the 16S rRNA gene do not draw identical pictures of the community profiles of fresh raw milk samples. Indeed, we found different, but overlapping lists of the top 10 predominant taxa in fresh raw milk for both regions. Moreover, compared to the V3-V4 region, the microbial profile using the V6-V8 region exhibited a higher richness, but lower alpha-diversity, indicating large dominance by few taxa. These results are consistent with previous studies which compared single or combined 16S rRNA hypervariable regions [61,62].

On the other hand, in unpreserved raw milk conserved at 4 °C for 5 days, we observed significant drops in richness and alpha-diversity measures for both hypervariable regions. This was accompanied by a highly significant shift in community structure again revealed by both variable regions. These results clearly show that storing raw milk samples at 4 °C is insufficient to stabilize the microbial community for up to 5 days, consistent with previous studies [8,9]. More importantly, our results confirm those from previous studies which reported the predominance of a diversified portion of the microbiota in refrigerated milk, including psychrotrophic and mesophilic bacteria [8,9]. Accordingly, we identified the genera *Lactococcus*, *Yersinia*, *Enterococcus*, *Macrococcus*, and *Leuconostoc* in V3-V4 sequences, and in V6-V8 sequences the genera *Acinetobacter*, *Macrococcus*, and *Rhodococcus* as biomarkers of 5-day stored raw milk, thus presumably spoiled raw milk. Once more, the identification of microbial taxa that characterize potentially spoiled raw milk during or after storage and transportation under refrigeration to dairy plants may provide different results depending on the sequenced region. Comparison of published results from partial 16S rRNA high-throughput sequencing is problematic due to the use of a wide variety of analytical techniques and bioinformatic pipelines. A consensus on the most appropriate hypervariable regions that best describe microbial communities, particularly for dairy associated bacteria, is yet to be established [63,64]. Identifying the best hypervariable region between V3-V4 and V6-V8 was, however, not the scope of the current study. Nevertheless, due to its greater coverage, the V6-V8 region seems more adapted for exploring the microbial composition of milk. For hypothesis driven microbiome studies aiming at characterizing or comparing different biological or environmental conditions associated with milk, the V3-V4 region would be preferred, as it appeared statistically more discriminative. However, the phylogenetic resolution provided by amplicon sequencing is a limiting factor in the microbial profiling and the evaluation of its dynamics in various ecosystems. With the recently developed approach that combines circular consensus sequencing from the Pacbio platform and the DADA2 algorithm to better classify long-read sequences up to sub-species level [65], targeting the 16S rRNA gene along its full length will lead to better resolution in species identification.

We assessed the immediate effects of preservative substances on milk microbiota before investigating their stabilizing ability. Our results showed that none of the preservatives adversely affected milk microbiota composition and structure once added to samples. This corroborates previous studies that showed that bacterial viability and recovery were not hampered within 24–48 h after azidiol was mixed with milk samples [9,22]. Our results indicate no negative interactions between the preservatives tested and the ability of PMA to target viable cells. Nevertheless, some biases were noted for a few taxa where abundance was significantly increased when AZ4, BR4, or AZDm treatments were applied, but not sufficiently to induce significant differences at the community level. The same trend was also noted for viable total bacterial loads determined by PMA-qPCR on AZ4 and AZDm treated milk aliquots. Even though the immediate bactericidal effect of treatment BR4 was significantly higher on LAB compared to other sub-groups quantified, the microbiota composition and evenness (alpha-diversity) in BR4-treated milk remained stable over the course of storage, as for that of other treated milk samples, regardless of the sequenced region. In contrast, the community structure (beta-diversity) varied significantly across time in BR4-treated milk, showing the highest variability in consistency measured through ICC analyses for all taxonomic levels. Our results showed that BR4 treatment is inappropriate to preserve milk samples intended for microbiome analyses. They confirmed those from Sierra et al. who used automated flow cytometry to evaluate the effect of bronopol on total bacterial counts in preserved goat milk [66]. The authors did not recommend bronopol as a preservation method preceding total bacterial counts because of its biocidal effect.

We also demonstrated, as have previous studies on other matrices [11,12,13,14], that freezing, particularly with a cryoprotectant such as DMSO [22], is the gold standard for preserving dairy milk samples. Indeed, we found that milk microbial community composition and structure were stable when frozen, supplemented or not with azidiol. However, our analyses on α- and β-diversity measures showed that although not always significant, there was a higher trend of differentiation between microbial communities across timepoints for DMSO-treated samples, compared to AZDm treatment. The tendency was more accentuated between the 10 and 30 days of storage. This indicates, as expected, that sample treatment with azidiol before freezing provides a better preservation of the microbial community in milk samples. To our knowledge, this is the first report on the preservative properties of azidiol combined with DMSO before freezing. We also found a better stability of the milk microbiota in AZ4-treated samples compared to DMSO-treated ones, specifically for up to 10 days post-storage. Our findings corroborate those of Martins et al. [16] who showed that the total bacterial counts in azidiol treated milk remained stable under refrigeration between 1 and 4 °C for up to 1 week. For large-scale studies in remote areas, sample refrigeration would be more easily and reliably achieved than freezing. Therefore, in this instance, treatment AZ4 would be preferred to AZDm, particularly to minimize the bacterial proliferation for short-term preservation before sample analysis. Besides bacteria, variation of fungal loads across time in treated and untreated samples were assessed by PMA-qPCR. We found that only treatments AZDm and DMSO were able to stabilize the total fungal community, while treatments AZ4 and BR4 were not able to stabilize them for at least up to 5 days. These latter treatments lacked freezing, which is well known for inhibiting fungal growth. In addition, the lack of any fungistatic or fungicidal substance certainly explains yeast and mold proliferation during milk storage. Several studies, as reviewed by Frey-Klett et al. [67], have demonstrated that fungi and bacteria occurring in the same environment can interact to favor their growth through diverse physical and chemical mechanisms. As fungal communities constitute an important part of raw milk microbiota [68], fungistatic substances (not fungicidal) should be used to limit their proliferation during sample storage. Indeed, preventing fungal growth in stored milk samples would logically improve azidiol efficiency, and ultimately allow long-term storage of AZ4-treated raw milk for microbial analyses.

Besides high throughput marker gene or shotgun sequencing, other omics approaches, as reviewed by Tilocca et al. [1], have been used to characterize the microbiota of dairy milk and products. Whether they involve gene expression analyses of microbial communities (metatranscriptomics), identification and quantification (meta-metabolomics) of their metabolites or proteome analysis (metaproteomics), they all depend on microbiome stability for consistent and reliable study results. Sodium azide, one of the main constituents of azidiol, has been successfully used to minimize bacterial growth during proteomic studies on distinct matrices such as milk [69] or urine [70]. Additionally, chloramphenicol, another constituent of azidiol, has been extensively used in studies characterizing proteome dynamics of microbial antibiotic resistance. To our knowledge, no incompatibility has been reported between sodium azide and chloramphenicol. Therefore, like the recently developed metaproteomic approach to decipher enzyme modulation by lysozyme treatment of Grana Padano cheese samples [71], future large scale studies might obviously benefit from the ability of the azidiol preservative to fix microbial communities in collected samples. However, depending on the matrix analyzed and the research objectives, optimization would be needed to ensure efficiency and practicality of the preservation method.

## 5. Conclusions

Using a “viability high-throughput sequencing” approach combined with viability-PCR, we demonstrated that a combination of azidiol and DMSO to preserve the microbiota of raw milk at freezing temperature was more efficient than simple freezing without a preservative agent. Interestingly, we showed that the microbiota in azidiol-treated milk was equally stable for up to 10 days at refrigeration temperature of 4 °C. Our findings suggest that in the latter condition, combining azidiol with a fungistatic substance would extend sample preservation time. Being among the most targeted hypervariable regions in high-throughput amplicon sequencing of the 16S rRNA gene, we proved that V3-V4 and V6-V8 regions do not always provide the same picture from the same microbial populations, highlighting caution in choosing the right region to sequence according to research objectives if short-read sequencing is to be performed. Based on the above findings, we encourage the use of azidiol for optimal conservation of raw milk microbiota intended for culture-dependent and -independent analyses in large-scale epidemiological or longitudinal studies.

## Figures and Tables

**Table 1:** PCR primers used for species or group quantification.

Microorganisms	Primer Name	Primer Sequence (5′-3′)	Gene	Amplicon Size	Reference
*Lactobacillus buchneri*	LBF2 LBR1	GAAACAGGTGCTAATACCGTATAACAACCA CGCCTTGGTAGGCCGTTACCTTACCAACA	16S rRNA	129	[30]
*Lactobacillus plantarum*	LacPlan1F LacPlan1R	AGGCGCGGCTGATGTCA CGCGATTGTCTTGGTTTTGTT	recA	68	[31]
Lactic acid bacteria	WLAB1 WLAB2	TCCGGATTTATTGGGCGTAAAGCGA TCGAATTAAACCACATGCTCCA	16S rRNA	407	[32]
Acetic acid bacteria	AQ1F AQ2R	TCAAGTCCTCATGGCCCTTATG CGCCATTGTAGCACGTGTGTA	16S rRNA	55	
*Enterobacteriaceae*	rplP 1F rplP 185R	ATGTTACAACCAAAGCGTACA TTACCYTGACGCTTAACTGC	rplP	185	[33]
*Pseudomonas* spp.	Pse435F Pse686R	ACTTTAAGTTGGGAGGAAGGG ACACAGGAAATTCCACCACCC	16S rRNA	251	[34]
Total bacteria	Uni334F Uni514R	ACTCCTACGGGAGGCAGCAGT ATTACCGCGGCTGCTGGC	16S rRNA	180	[35]
Total fungi	ITS1f 5.8S	TCCGTAGGTGAACCTGCGG CGCTGCGTTCTTCATCG	rRNA	300	[36]

## Supplementary Materials

The following are available online at https://www.mdpi.com/2076-2607/8/3/368/s1, Figure S1: Heat map showing the distribution, prevalence, and abundance of taxa at the species level in fresh and 5-day stored unpreserved raw milk based on the V3-V4 dataset. Figure S2: Heat map showing the distribution, prevalence, and abundance of taxa at the species level in fresh and 5-day stored unpreserved raw milk based on the V6-V8 dataset. Figure S3: Taxonomic markers characterizing fresh (colored red) versus 5-day stored (colored green) raw unpreserved milk as depicted by high-throughput sequencing of the 16S rRNA gene targeting the V3-V4 (A) and V6-V8 (B) hypervariable regions. Figure S4: Changes occurring in the microbial composition and structure of raw milk once subjected to preservatives. Figure S5: Box plots illustrating multiple comparison tests between storage timepoints of preserved raw milk as described by amplicon sequencing of the V3-V4 variable region. Figure S6: Temporal stability of microbial communities in preserved raw milk across 30 days of storage as described by 16S rRNA gene sequencing of the V6-V8 region. Figure S7: Box plots illustrating multiple comparison tests between storage timepoints of preserved raw milk as described by amplicon sequencing of the V6-V8 variable region. Figure S8: Community instability and taxa dynamics in unpreserved and preserved milk samples over the storage time as depicted by high-throughput amplicon sequencing of the 16S rRNA targeting the V6-V8 region.
