# Supplementary material for: Optimization of Preservation Methods Allows Deeper Insights into Changes of Raw Milk Microbiota
Source: Microorganisms. 2020 Mar 5;8(3):368. doi: 10.3390/microorganisms8030368 (PMC7142718; doi:10.3390/microorganisms8030368)
Supplement: Supplementary file 1 [file microorganisms-08-00368-s001.pdf]

SUPPLEMENTAL MATERIAL

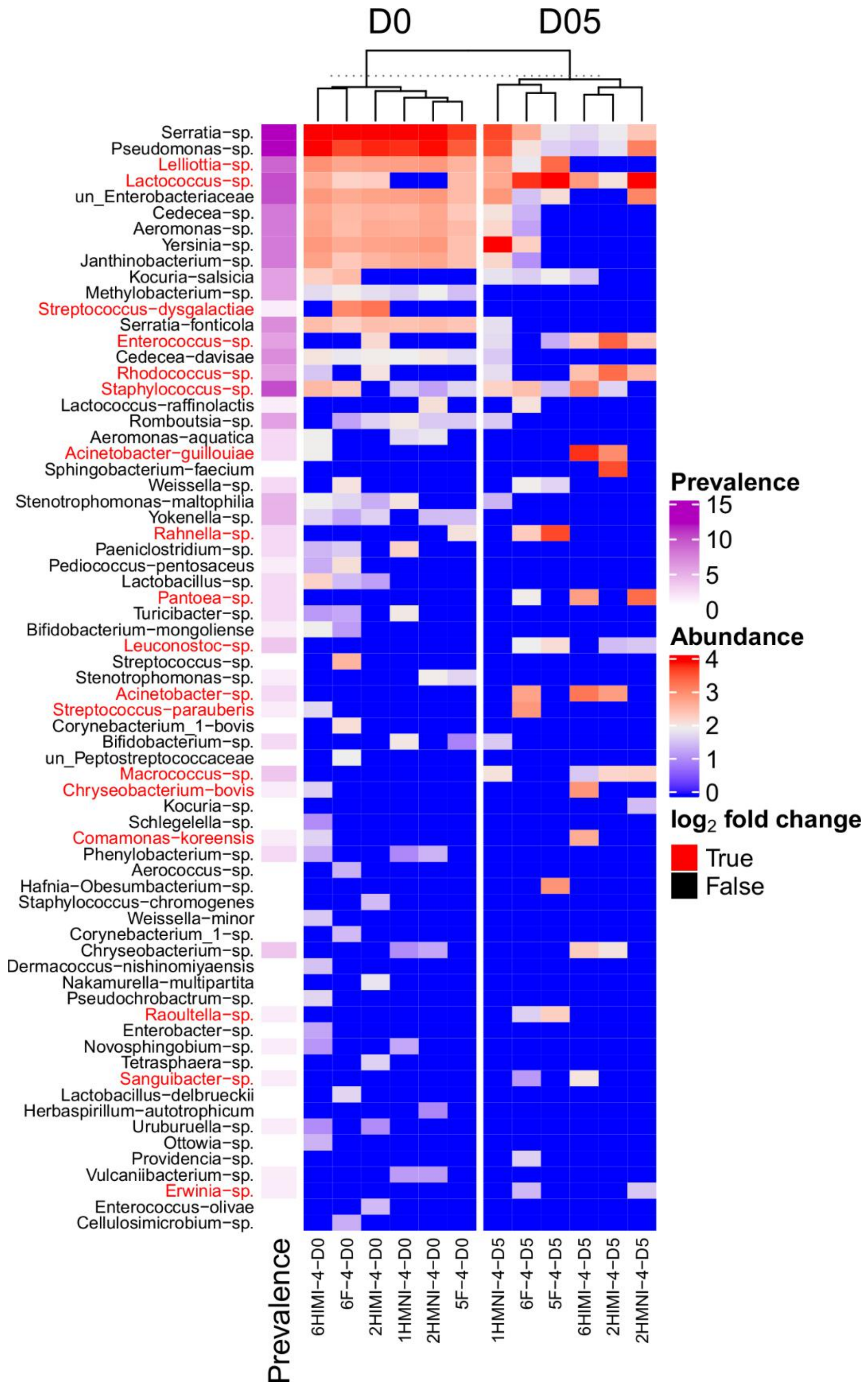

Figure S1: Heat map showing the distribution, prevalence, and abundance of taxa at the species level in fresh and five-day stored unpreserved raw milk based on the V3-V4 dataset. Taxa coloured red are those that underwent log2-fold change during the storage.

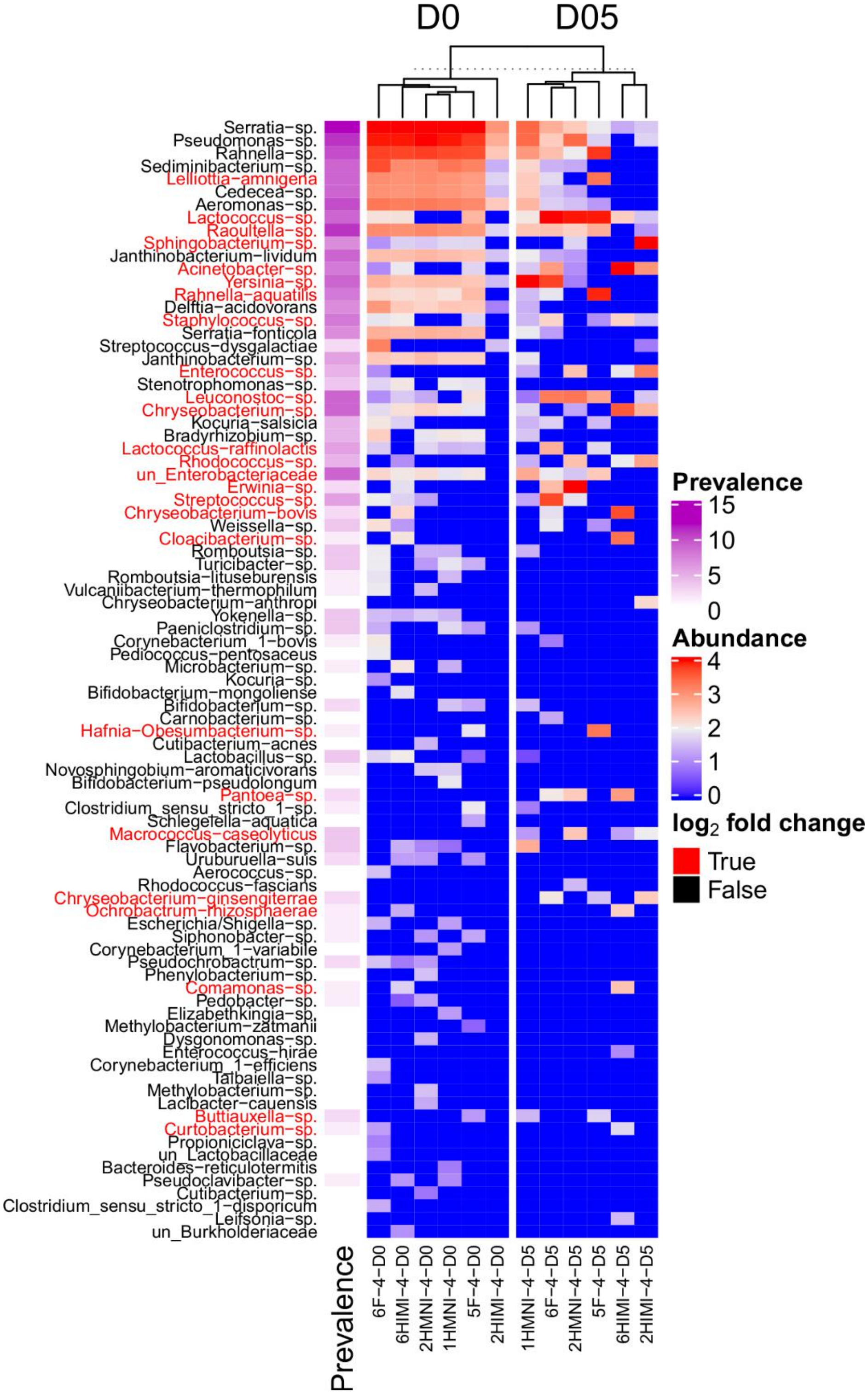

Figure S2: Heat map showing the distribution, prevalence, and abundance of taxa at the species level in fresh and five-day stored unpreserved raw milk based on the V6-V8 dataset. Taxa coloured red are those that underwent log2-fold change during the storage.

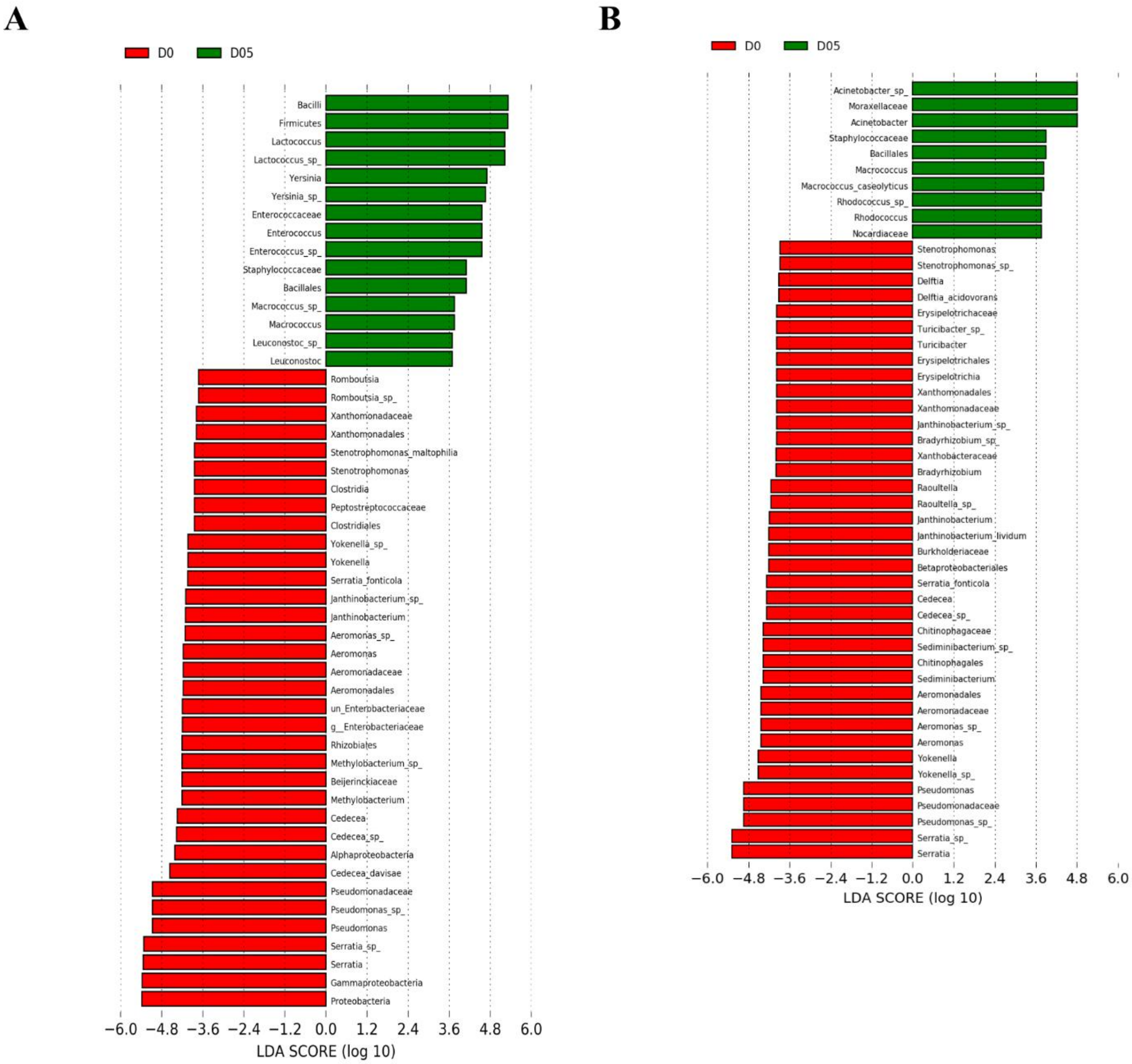

Figure S3. Taxonomic markers characterizing fresh (coloured red) versus five-day stored (coloured green) raw unpreserved milk as depicted by high-throughput sequencing of the 16S rRNA gene targeting the V3-V4 (A) and V6-V8 (B) hypervariable regions.

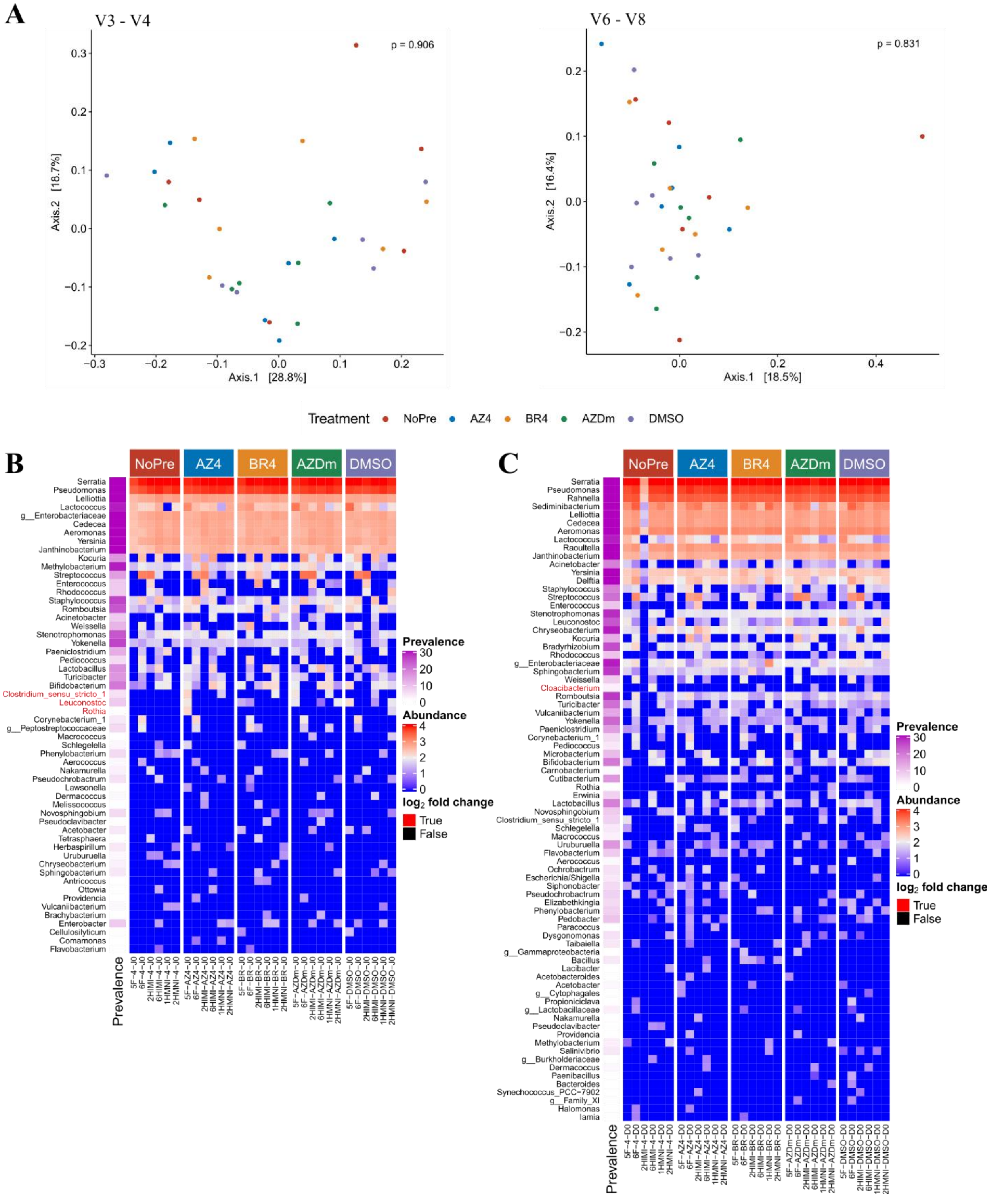

Figure S4. Changes occurring in the microbial composition and structure of raw milk once subjected to preservatives. Treatments include NoPre (untreated raw milk), AZ4 (raw milk treated with azidiol), BR4 (Bronopol-treated raw milk), AZDm (Raw milk treated with a mixture of azidiol and dimethyl sulfoxide), and DMSO (raw milk treated with dimethyl sulfoxide). (A) Principal coordinate analysis on unweighted UniFrac distances based on the V3-V4 (left) and

the V6-V8 (right) datasets. (B-C) Prevalence and abundance of taxa (coloured in red) that underwent ≥ log2-fold changes based on the V3-V4 (B) and V6-V8 (C) datasets respectively.

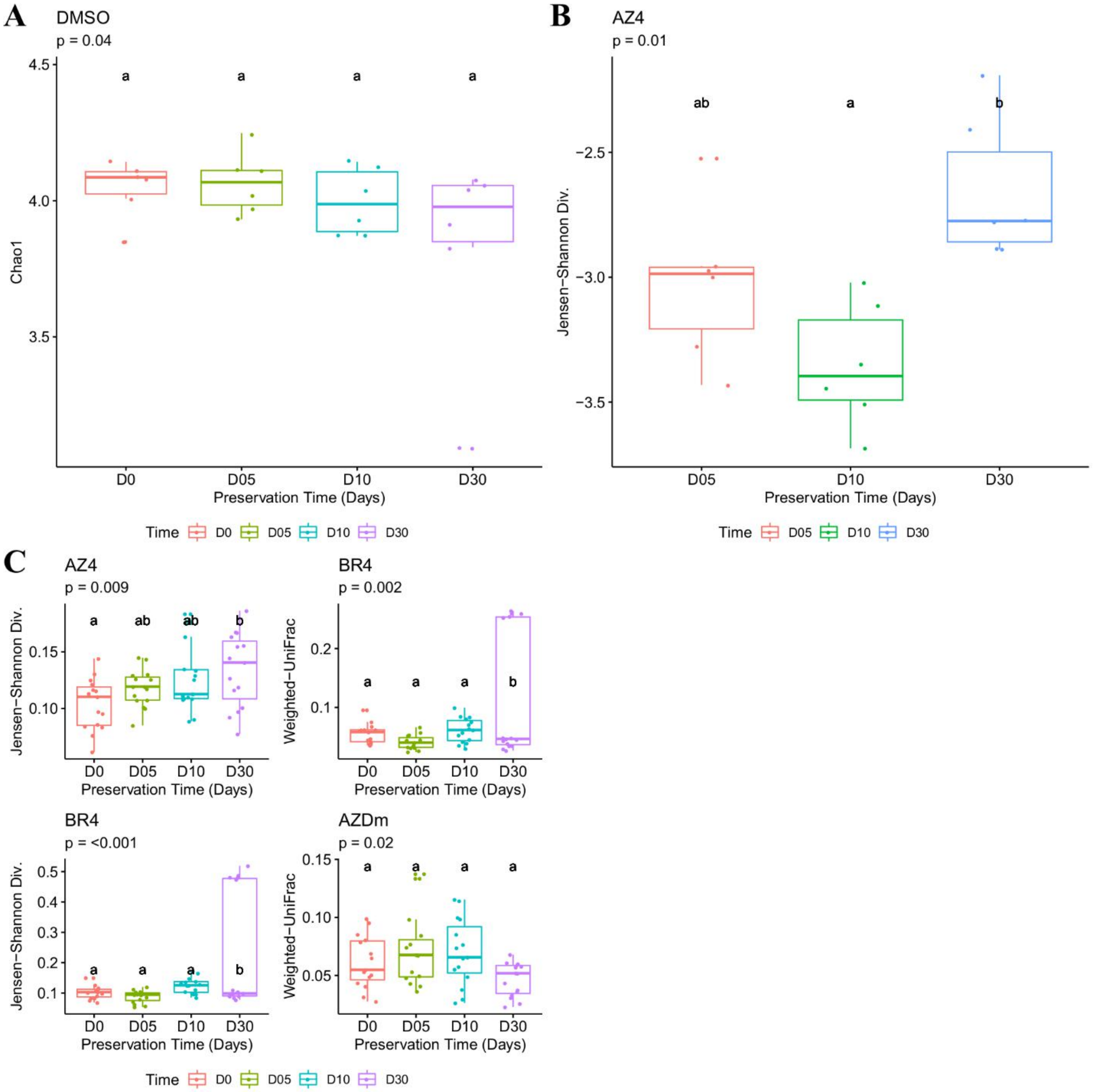

Figure S5. Box plots illustrating multiple comparison tests between storage timepoints of preserved raw milk as described by amplicon sequencing of the V3-V4 variable region. Letters above a box indicate significance group. Timepoints with different letters were significantly different. P-values provided are derived from the corresponding lme analysis shown in Fig. 5 of the main text. (A) Multiple comparisons based on the Chao1 estimates analysed by lme modelling and visualized in Fig. 5A. (B) Multiple comparisons based on the Jensen-Shannon divergence measures computed within aliquots of the same sample for treatment AZ4 as illustrated in Fig. 5B. (C) Multiple comparisons based β-diversity measures computed between aliquots of all samples for treatments AZ4, BR4 and AZDm as illustrated in Fig. 5C.

NoPre: No treatment, AZ4: Azidiol, BR4: Bronopol, DMSO: Dimethyl sulfoxide, AZDm: Azidiol & DMSO.

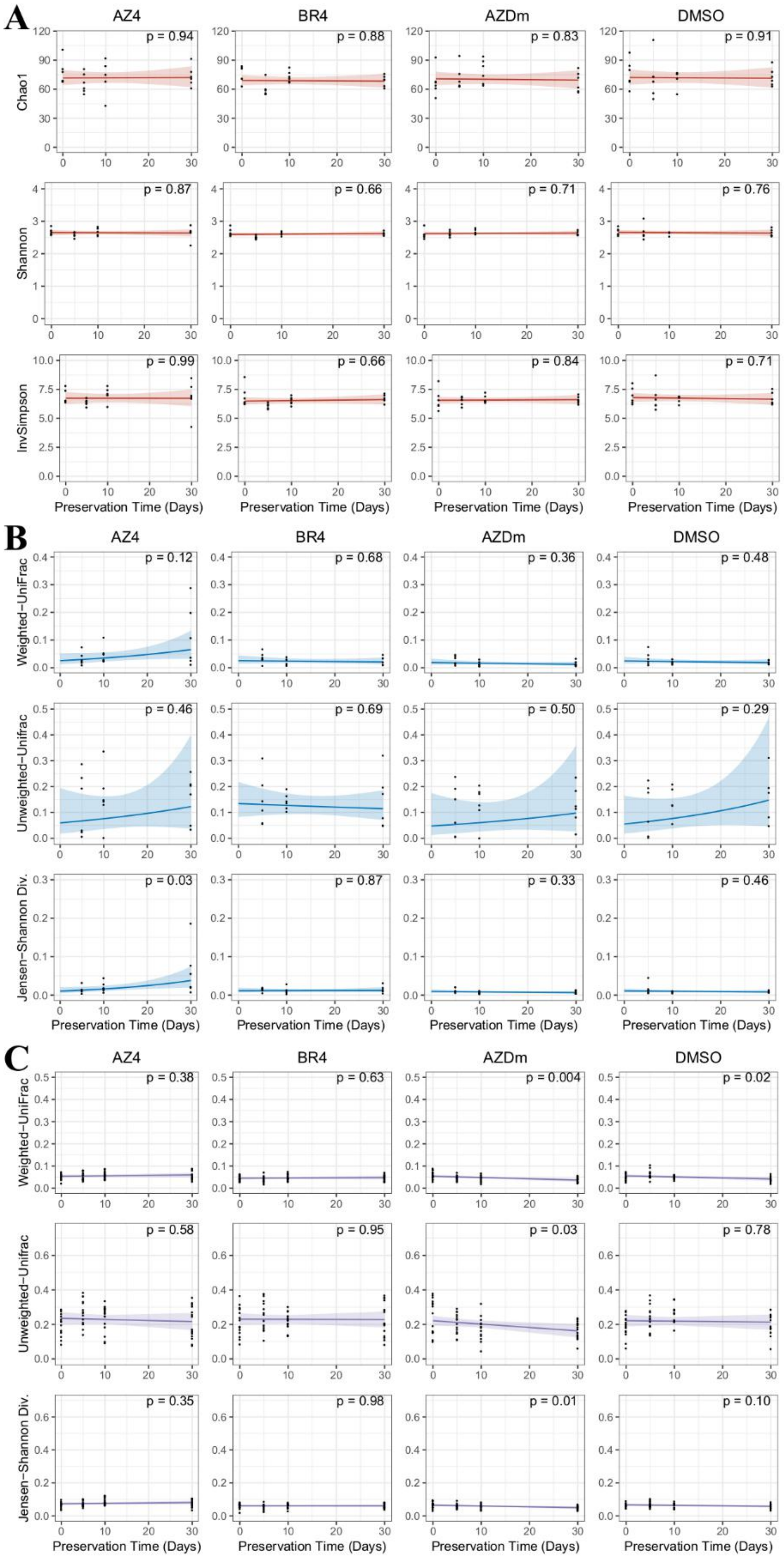

Figure S6. Temporal stability of microbial communities in preserved raw milk across 30 days of storage as described by 16S rRNA gene sequencing of the V6-V8 region. (A) Variation in alpha diversity measures during the storage of treated raw milk. For Chao1 estimates, Shannon, and InvSimpson indices, salmon lines represent linear mixed-effects

fit against the storage time and the 95% confidence interval is shaded. (B) Diversity trends between samples from the same farm at consecutive timepoints. Blue lines represent linear mixed-effects fit against the storage time and the shade the 95% confidence interval. (C) Diversity trends between samples from different farms at consecutive timepoints. Purple lines represent linear mixed-effects fit against the storage time and the 95% confidence interval is shaded.

NoPre: No treatment, AZ4: Azidiol, BR4: Bronopol, DMSO: Dimethyl sulfoxide, AZDm: Azidiol & DMSO.

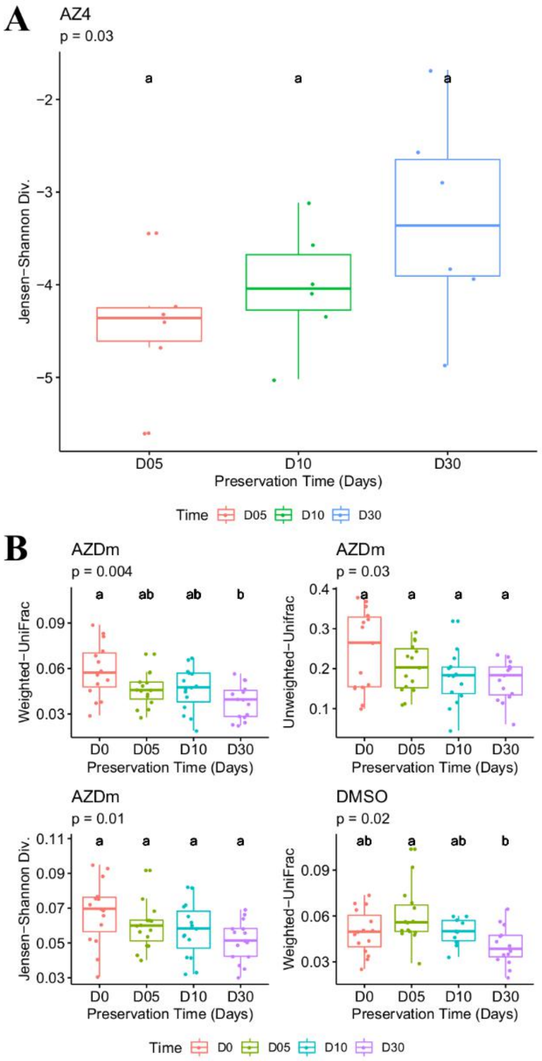

Figure S7. Box plots illustrating multiple comparison tests between storage timepoints of preserved raw milk as described by amplicon sequencing of the V6-V8 variable region. Letters above a box indicate significance group. Timepoints with different letters were significantly different. P-values provided were derived from the corresponding lme analysis shown in Fig. S5. (A) Multiple comparisons based on the Jensen-Shannon divergence measures computed within aliquots of the same sample for treatment AZ4 as illustrated in Fig. S5-A. (B) Multiple comparisons based β-diversity measures computed between aliquots of all samples for treatments AZ4, BR4 and AZDm as illustrated in Fig. S5-C.

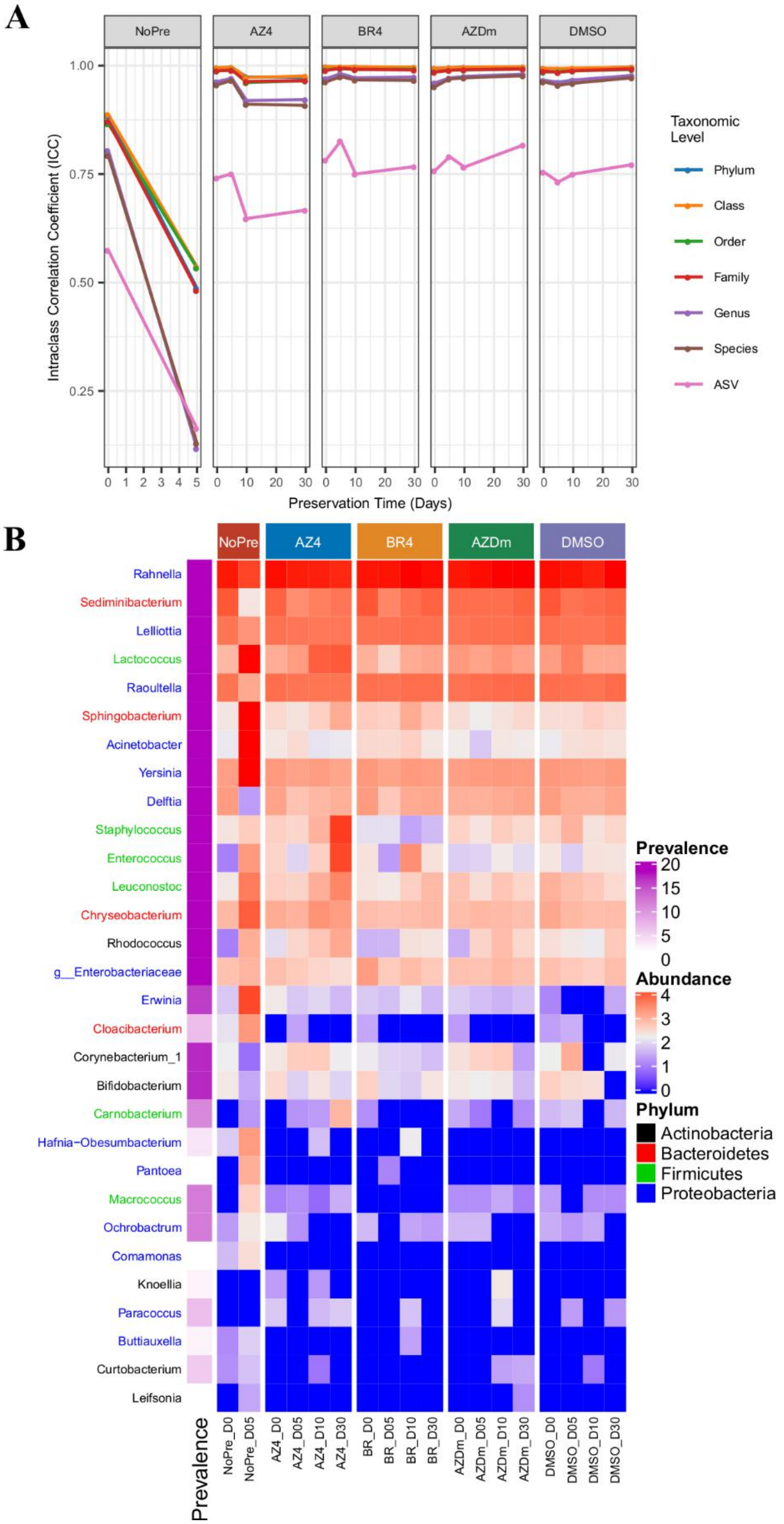

Figure S8. Community instability and taxa dynamics in unpreserved and preserved milk samples over the storage time as depicted by high-throughput amplicon sequencing of the 16S rRNA targeting the V6-V8 region. (A) Intraclass correlation coefficients computed for all the taxonomic levels between samples from different farms and plotted

against storage time for preservation-free and preserved samples. (B) Prevalence and abundance of taxa that underwent ≥ log2-fold changes in unpreserved and preserved raw milk during storage. Each taxon at the genus level is coloured by its corresponding phylum.
